# Philadelphia County Medical Society

**Published:** 1892-06

**Authors:** 


### Society Proceedings.

### PHILADELPHIA COUNTY MEDICAL SOCIETY.

THE President, JOHN B. ROBERTS, in the Chair.

### PNEUMONIA AND ITS TREATMENT.

BY HIRAM CORSON, M. D.
Plymouth Meeting, Montgomery County, Pa.

[*Read April 13, 1892.*]

What is pneumonia ? That question correctly answered, we will be prepared to treat it. Professor George B. Wood, of the University of Pennsylvania, one of the most accurate describers of disease and a practitioner of great eminence, to whose graphic descriptions of disease many of you have listened, says in his *Practice of Medicine*, that

Pneumonia has three stages, and is universally applied to inflammation of the lungs. In the first stage, the lungs are merely engorged with blood, and the air-cells partly filled with a sero-mucous, somewhat bloody effusion. In the second stage, a plastic extravasation has taken place, and the cells are filled with more or less concrete and bloody lymph. In the third stage, the place of the plastic secretion has been supplied by a purulent fluid.

Dr. Wood was a truthful man, had great opportunities in the Philadelphia Hospital to see the condition of the lungs in the different stages described by him, for they may all be present in a lung at the same time, when the disease continues to embrace fresh portions of it. In that I doubt not he is correct. But as the subject which now engages our attention is one of great importance, I beg you to bear with me while I quote from another author. Dr. G. R. Martine, of Glens Falls, N. Y., in a paper read before the American Medical Association in June, 1889, published in its journal September, 1889, — a paper remarkable for its accurate description of the disease as it exists in cases involving much of the lung, and for erroneous deductions in relation to treatment,— thus speaks :

The first abnormal symptoms, after the premonitory chill, is the quickening of the pulse and the consequent increased flow of arterial blood. Now, if we could take a microscopic view of the minute arterial ramifications in the lung structure, we would discern a distention in the caliber of the air-vessels in order to accommodate the augmented flow of blood ; and if we would then glance at the veins we would observe the plasma layer rapidly filling with white corpuscles, and the walls of the veins, irritated by the friction of increased circulation, would exhibit, here and there, white corpuscles adhering to their tenacious sides and finally penetrating their walls. A glance at the capillaries would show not only the white, but also the red corpuscles forcing their way through the overstrained capillary walls until the surroundings became engorged by their extravasation, and hepatization has commenced.

Now, if we bear in mind the condition of the lungs as described by the writers, we must believe that pneumonia is first a congestion, then an inflammation, and if that be not arrested suppuration takes place. Dr. Wood says: "It has been doubted whether a cure is ever effected in this last stage."

We are now brought face to face with the disease. Is there anything we can do to remove the congestion? The patient has been sick less than twenty-four hours, he is not yet expectorating bloody sputa, and many of the opponents to blood-letting say this disease cannot be aborted; that, like measles, it has a course to run, despite all interferences; that it is a constitutional disease, and that if we can prevent the congestion from being too suffocative, the inflammation from involving too much of the lung, we may let it run its course. The members of our profession are divided into two classes: those who believe that the disease may be aborted, *i. e.*, arrested in the congestive stage if early called, and in the stage of inflammation before suppuration has taken place; and the much more numerous class, who believe that it cannot be aborted; that a case, even if it be in its incipiency, must go on,—should be watched by the physician, and shorn of its power to take life, but be allowed to go on until a crisis is reached after a positively fixed number of days. Physicians of the first class aim to relieve the overloaded and suffering lung by the abstraction of blood by means of the lancet, from the veins of the arm; and those of the second class, who boast that they "never bleed" in any disease, depend on giving relief by reducing the increased action of the heart with medicines which have that effect. But the members of this latter class are greatly divided in opinion as to the proper medicine to be used to hold the heart in check; what one regards as being very useful and safe, another considers dangerous,—more dangerous than the disease, and withal inefficient,—yet all speak from experience, each one with his remedy. Experience is a harmful thing as a reason for continuing to practise a certain course, unless it has been successful experience. It reminds me of a case: I had attended a woman in her confinement, and as she expected to have the child raised by hand, as the phrase goes, and had read my paper on Food for Infants, she desired me to speak to the nurse about her mode of feeding the child ; so, when she appeared, I said : " Nurse, do you know how to feed a child so as to rear it without the mother's milk ? " " Oh, yes ! I have had experience in doing that ; my sister had three that I had to raise that way. " " Well, how did they come on ? " " Oh ! very well ; one of them lived until it was six months old." Who among us but can look back on measures which we practised, and which we believed indispensable because we had proved them, as we thought, by a long experience, but which we now see were not only useless but injurious. This, then, successful experience in the treatment of pneumonia is what we should follow. The absence of success, the fearful mortality attending the arterial sedative practice of the " no blood to spare " party ; the immediate relief, the successful arrest of pneumonia by blood-letting, during a whole century, should cause the opponents to venesection to cease their abuse of those whose successful experience in the use of it has been testified to by some of the most eminent men of this city and this country. A few weeks ago I was told by a gentlemen, holding a high State office, that a physician of whom he inquired concerning the recent death of a prominent man, replied: " Oh ! he was murdered by blood-letting." Does this man, who speaks so boldly about a measure of relief, of which he knows nothing from actual experience, who never used the lancet in his lifetime, denounce Prof. George B. Wood, Physic, Parrish, and the elder Hartshorne, Chapman, Samuel George Morton, Ezra Michener, Prof. Henry H. Smith, the Atlees — John and Washington — Traill Green, Henry Hartshorne, Jackson, of Northumberland, N. S. Davis, Prof. S. D. Gross, and hundreds of others who could be named, as conscientious as himself and far more truthful ? We have borne this stereotyped abuse long enough. Look at the terror spread over the country now, when these numerous deaths of the best of our people are every day announced, though all were in the hands of practitioners skilled in arterial sedation. Let us now see what the opponents of blood-letting resort to, to save the sufferers from death in this disease. From what I have learned of the present teachings in our colleges, and by conversations with practitioners, the main object is " to keep down the pulse "— that is the phrase. To do this, the most approved medicine is the fluid extract of veratrum viride.

"The object aimed at," says Dr. Martine, "is to hold the pulse below eighty;" and adds, "*that* is not only what should be done, but what *must* be done to save life." I know full well *that* is not necessary. Scores of times, after I have bled freely, with great relief to the patient and arrest of the disease, the heart, though tamed by the bleeding, continued its pulsations at from 80 to 100 per minute, or sometimes even more, for two or three days, and yet the convalescence went on. Again, he says: "With what remedies do I hold the pulse at this point (below eighty)?" and adds "veratrum viride seems to have served one best." To this, Dr. H. A. Hare, who was present at the reading, replied: "A great mistake is made in saying use cardiac sedatives in pneumonia without recognizing the fact that they are to be used only in the first stage, before congestion has gone on to consolidation; the man who gives such drugs at the middle or end of an attack of pneumonia, might as well stab his patient. Digitalis is to be used at such times." It therefore appears that it is only in the congestive stage that veratrum is to be used. If this be true,—and doubtless it is based on experience,—how many patients have been sent to their graves by this much-used medicine! for well do we all know that it is the most used of all arterial sedatives to keep down the pulse. And even Dr. Martine has peremptorily declared that it must be kept down, and that veratrum viride seems to have served him best. But it is not only Dr. Martine who disregards the stage of the disease, and uses it in all stages if the pulse be above eighty. I have long believed that nearly all the cases of pneumonia which terminated in death within four days, and I know several such, were hurried there by this potent and dangerous drug. Dr. I. E. Atkinson, of Baltimore, followed Dr. Hare, and said that "the use of veratria in the treatment was not new; it had been several years under trial, and had not received general acceptance." If Dr. Hare's utterances be true, then veratrum viride can scarcely be used at all without killing the patient, for a physician is seldom called until the congestion has passed into the second stage, when, according to Dr. Hare, you might as well stab the patient as to give it. How unerring must be the diagnosis, in order that this medicine may be given without risking the life of the patient. I have inquired of many of the "no blood to spare" class, and have rarely found two that have the same treatment. Those who use aconite are afraid to use veratria, while the latter regard aconite as being more dangerous and less efficient. Those, too, who use digitalis are afraid of the two drugs just named, and those who give two drops every two hours denounce others who give large doses of it (ten to twenty drops, frequently repeated,) as pursuing a most dangerous practice. Judging from the accusations made by themselves against each other, what safety is there to the patient ? The answer comes in saddest tones from homes made desolate by dangerous arterial depressants. To the two classes of opponents to blood-letting spoken of, there are others which should have a passing notice : first, those who rely on the use of sulphate of quinine to keep down the pulse and to diminish the heat of the body. While quinine is, in malarial diseases, an unrivaled medicine and has done valuable service, it is useless, if not really most injurious, in the treatment of pneumonia and other inflammatory diseases. We should feel greatly indebted to Dr. Horatio C. Wood for the experiments which proved to him that even very large doses of that medicine cannot hold the temperature of the body at a low figure. There was so much stress laid on the importance of preventing the heart failure, by keeping down the temperature by means of quinine above all other medicines, because of its tonic powers, that Dr. Ripley, the two Drs. Jacobi, and three other physicians of New York City, made careful and repeated experiments to determine the value of the drug in that respect, and demonstrated beyond cavil that it is never useful, and often greatly objectionable—really injurious—in the treatment of pneumonia. I have often felt exceedingly thankful to Dr. Wood and the New York doctors for their careful and effective labors in that direction, and greatly amazed that, in the face of the assertions that quinine is useless in pneumonia to effect the purpose for which it is used, some persons still persist in its use. Many lives have thus been lost. The second class of those who fraternize with the arterial sedative practitioners is composed of the whiskey or stimulant practitioners,—the physicians who see typhoid and blood-poison symptoms in almost all diseases. I will spend but little time with them. They are belated people, clothed in, and proud of, the cast-off garments of progressive physicians. Let us look calmly at this subject. Are there physicians here who can say that there are medicinal properties in whiskey or in brandy which, in either large or small doses, warrant us in trusting the life of a patient to their action ? It is a serious thing to experiment with human life. No means but those which have proved successful in numerous cases should be used when our patients are struggling for breath, and death hovering over them. There is nothing more saddening to me in the sick-chamber than to see a physician forcing alcoholic drinks on the dying patients, and yet it has been done countless thousands of times, and now ofttimes they are used in the very earliest stages of pneumonia. Dr. N. S. Davis does not believe them essential in any disease. If, then, arterial sedatives, quinine, and alcohol, are not adapted to relieve the congestion and inflammation of the lung, which constitute pneumonia, is there any remedy for that now fatal disease ? We know that there is, but not from an experience like that of the nurse already named, nor of another one, greatly experienced, of whom allow me to speak. I was called to a child, lying on the lap of a friend, "because she had had experience with such cases." I said, "The child seems very sick." "Yes," she replied, "it has summer complaint, and it will die." "How do you know it will die ?" "Oh ! I have had experience ; I have had ten of them with it." "All your own children ? did they all die ?" "Yes." "Did you have the same doctor for them all ?" "Yes." "Well, your experience is worth nothing." I treated the child, and, to her disgust, it was soon well. Her experience is a fair type of the experience of those physicians who go on with the same fatal treatment, lose one patient after another, and speak of "experience" in treating the disease.

But now a word about those who "murder" their patients by bleeding them. What are the objections urged against blood-letting ? and by whom ? The first objection is, "No one has any blood to spare." If this be true, then it embraces all other objections, and none need be named. This certainly means that in health, as well as in disease, the loss of even a small quantity of blood would be injurious to the loser of it. It means more than that—means, as is boldly asserted, that it is a permanent injury to the body, the bad effect of which is seen in the permanently weakened system of the person. Thousands of facts disproving this, and which are daily before our eyes, count for nothing. One would suppose that if half a dozen, or even one, of the respectable, truthful men here should declare that he had often bled persons in various diseases with the greatest benefit to them, nothing more would be needed to prove the falsity of the declaration, "no blood to spare"—a cry so senseless, so false, that no decent man should utter it.

The very first act of these objectors after the birth of a child, in cases attended by them, is deliberately to cut the cord and waste two ounces of blood that ought to have passed into the body of the child.[1] But there is another objection urged, viz., that even in the earliest stage of the disease under consideration, though some relief may be obtained, no blood should be taken, lest it should leave the patient too weak to resist the exhaustion of a later stage,—leave him in a condition in which even whiskey and rich food would, though *pushed*, not be able to save him from death.

Is it, then, injurious to take blood from a pneumonic patient? This is the cry and the charge of those who have never seen a patient bled, and yet who have known its safety and value to be testified to by some of the most eminent physicians who have ever lived in this century or preceding ones. In my last four papers, published in the *Medical and Surgical Reporter*, I have given a few cases reported by the Conshohocken doctors, which were bled freely, and were so successful that opposition to venesection should be silenced in those who read them. I will now speak of some seen by myself, and in doing so will present some views which I have long held:

On February 16, 1887, I was called to see a physician two days after an acute, serious attack of pneumonia. I found that he was under treatment by one "who never bleeds in any disease." He was expectorating the bloody "rust-colored, prune-juice" sputa, a quantity of it being in the basin; had great oppression and that peculiar sense of great weakness so constant an attendant in pneumonia when the obstruction to free breathing exists. I had to wait nearly three hours for the return of the physician, and when he did come he was opposed to bleeding, and refused to accede to it, but proposed that the patient choose whether or not he would be bled. The patient knew well our different modes of treatment, and promptly said, "I will be bled." I asked the doctor to remain, and, slipping another pillow under the sick man's head, I drew blood until I found the pulse yielding in *force*, and knew that faintness was approaching. Withdrawing the pillow to lower the head, I closed the vein and took a seat. In about ten minutes I inquired, "How do you feel?" "I am much relieved; I think it will do me great good." This was at noon. Went again in the evening; he was pretty comfortable, breathed much more easily, and had not coughed up the least particle of blood or rusty sputum since the bleeding. The distended blood-vessels were relieved of their fulness, and "the slow exudation from the inflamed vessel" spoken of by Dr. Wood was no longer forced through their coats. Would one or twenty doses of arterial sedatives have produced that effect? Having bled my patient, what next did I do? Nothing, but told him that I would see him in a few hours.

1. See Transactions of the *Pennsylvania State Medical Society* for 1872, p. 154.

And here let me say that, in the case already alluded to, when I saw by the papers that he had been bled, I said, "in all human probability he will die!" "Why?" was asked. "Because he was *timidly bled by one unused to venesection and fearful of it*, and because the other depressing, arterial, sedative treatment will follow it, and the patient will suffer from their poisonous, depressing effects."

To return to my case: When I saw the patient a few hours afterward I found him pretty comfortable. I directed one Dover's powder, to be repeated in the night, if need be, because of pain. Next day a mild diaphoretic was administered.

Two days after this a physician who, as a neighbor, had given much attention to his sick friend, asked me: "How do you find your patient?" I said: "He is well; he needs only a few days in bed to regain his strength." I left for home, and he went to the patient and said to him: "The doctor told me just now that you are well. What was it that cured you? was it that blood that came from your arm?" He replied: "The blood did not come from my arm; it came from here," laying his hand on the affected side. "I felt it going from here as it went into the basin." How strong this testimony, given by a physician who felt in his own person the relief obtained by venesection! How confirmatory, too, is this of the testimony of Dr. Gross, the elder, who, in a discussion, had in the State Medical Society illustrated the effect of venesection in this way:

Should a man have an inflammation of the conjunctiva, and the capillary vessels be so injected that the blood was of a deep redness; and then he, being in a sitting posture, should be bled largely, the blood would be drawn from the capillaries, and the redness disappear. Just so does it draw the blood away from the capillaries of the congested and inflamed lung.

Veratrum viride in doses large enough to keep the pulse below eighty, would not produce the least relief to the distended capillaries. Having bled the patient, if in a few hours there is not much relief, he may be bled again, if needful, and even more than once. But what of local measures? Do I approve of the poultice or the pads of cotton to envelop the chest? No. Two years ago, after my criticism of Dr. Wells's paper was published, an aged physician regretted that I had not spoken of the value of "the blister" over the affected part. The subject before me was only that of Bloodletting: Its Value or Danger. It is the same now, but I may add here that the application of cloths dipped in ice water is to the patient the most agreeable application that I have ever used.

Despite the great length of this paper, I beg of you to hear the utterances of one of our most eminent men to his students at his clinic in the University Hospital, and published in the February number of the *International Medical Magazine*, pp. 43, 45. Dr. William Pepper stated to his pupils at the clinic, that

The man of twenty-eight years had been well until ten weeks before his admission to the ward, and had during that time been treated by several physicians. On admission, November 5th, his distress was extreme; he was unable to lie down or recline, and was obliged to remain constantly in a sitting position, but did not get relief from leaning forward.

Then follows a long and interesting account of the illness and treatment; after which follows:

On November 7th, the third day of his stay at the hospital, the symptoms were so alarming, with deep cyanosis, labored action of the heart, orthopnea, and high fever, that I had him bled from the arm to the extent of twenty ounces. The good effect of this was immediate, and although cyanotic symptoms returned to some extent on the following day, a material improvement dated from the time of the venesection.

Again, the learned professor at great length gives his views of the case, and before closing his paper, remarks, January 5th:

He has continued to do well, and is now thoroughly convalescent. Before closing, I would call your attention to the extraordinary effect which followed the abstraction of blood. All of us who saw his condition before the bleeding, and watched the immediate effect of this, were satisfied that his life was saved thereby. I doubt if any other remedy could have acted so promptly and efficiently. It was the observation of such striking results, when bleeding was used in suitable cases, that gradually led our medical forefathers to rely upon it more and more in grave crises, until its occasional and legitimate use degenerated into almost promiscuous abuse. It is one of the tasks set before clinical medicine today to indicate with the greater precision, rendered possible by our improved methods of investigation and more full knowledge of the natural history of disease, the exact conditions under which this most powerful remedial measure is to be adopted.

Such are the recent utterances of the able editor of *A System of Medicine by American Authors*, in which is a paper by Alfred Loomis, M. D., Professor of Practice in the University of New

York, who believes "pneumonia a constitutional disease, with a local manifestation," and regards blood-letting as a dangerous plan of treatment. Such, too, has been the views taught to large classes of students by Prof. Pepper, until he made trial of the remedy. I sincerely hope that Prof. Loomis, too, will make a trial of it hereafter, and be induced, like Dr. Pepper, "to doubt that any other remedy could have acted so promptly and efficiently."

I cannot be too greatful to Prof. Pepper for his valuable testimony in relation to venesection, and for the hope which he has given us, that hereafter he will not withhold this potential means of relief from those suffering in the grasp of this (now) too fatal affection. It is one of the most remedial diseases when properly treated; but when managed by arterial sedatives and their aids, stimulants and excess of food, a most fatal one. More than sixty-five years of careful, anxious observation of the effects of blood-letting in pneumonia have proved to me that it has no rival as a remedy for that disease.

